# Treatment of a Large Skull Defect and Brain Herniation in a Newborn With Adams-Oliver Syndrome

**DOI:** 10.7759/cureus.7047

**Published:** 2020-02-19

**Authors:** Kim M Wehrens, Frank De Jongh, MP Ter Laak, EM Cornips, RRWJ Van der Hulst

**Affiliations:** 1 Plastic Surgery, Haaglanden Medisch Centrum, The Hague, NLD; 2 Neurosurgery, Maastricht University Medical Center, Maastricht, NLD; 3 Plastic Surgery, Maastricht University Medical Center, Maastricht, NLD

**Keywords:** adams-olivers syndrome, congenital disorder, plastic surgery, conservative treatment, aplasia cutis congenita

## Abstract

Adams-Oliver syndrome (AOS) is a rare congenital disorder characterised by a wide variety of clinical expression ranging from the occurrence of aplasia cutis congenita (ACC), transverse limb defects, and cutis marmorata telangiectica to extensive lethal anomalies. In this article, we present the conservative and surgical management of a male newborn infant diagnosed with AOS. Surgical treatment included wound management, the removal of protruding brain, and treatment of cerebrospinal fluid (CSF) leakage. After spontaneous reepithelization of the wounds, conservative treatment was chosen instead of reconstruction with an occipital flap; this was continued until the total healing of the dermal defect after eight months, during which the patient was continuously treated with antibiotics. At 17 months, the child was in good physical condition with a three-month development delay in comparison with infants of his age and no evidence of neurological deficit.

## Introduction

Adams-Oliver syndrome (AOS) is a rare congenital disorder characterised by a wide variety of clinical expressions. This ranges from the occurrence of aplasia cutis congenita (ACC), transverse terminal limb defects (TTLD) and cutis marmorata telangiectatica congenita (CMTC, 19%) to extensive lethal anomalies to the central nervous system (CNS, 23%) and congenital heart defects (23%) [1,2].This syndrome was first described in 1945 by Adams and Oliver [3]. Most cases of AOS are assumed to be autosomal dominant with reduced penetrance and variable expression, however, in some cases there appeared to be an autosomal recessive inheritance pattern. The common occurrence of cardiac and vascular anomalies suggests a primary defect of vasculogenesis, although the molecular basis of this disorder still remains unknown [4-8]. Six causative genes (NOTCH1, DLL4, DOCK6, ARHGAP31, EOGT, and RBPJ) have been identified [9-11].

Several cases of ACC have previously been described; some of them were associated with the AOS.

We report a unique case of a male newborn infant with an exceptionally large congenital scalp and skull defect exposing the dura, and herniation and active bleeding of the brain, owing to AOS. Furthermore, the patient had small TTLDs (minimal brachydactyly).

Management of skull defects resulting from cutis aplasia remains controversial, probably because of the low prevalence, which has been estimated at 1 per 10.000 live births [12]. Both surgical intervention and conservative management, or a combination of the two have been described in the literature [12-19]. As recent literature has highlighted the potential, serious risks of nonsurgical management of large extended congenital skull defects [12], we decided to report our case in favour of a conservative approach.

## Case presentation

Case presentation

A male newborn infant was born through an acute caesarean section at 39 weeks gestation at another medical hospital. He was the second child of phenotypically normal, non-consanguineous parents. The pregnancy was complicated by intrauterine growth restriction (IUGR) (<P10). Antenatal ultrasound at 32 and 35 weeks’ gestation at the University Hospital in Maastricht revealed a delayed growth of the head compared to the growth of the body. Brain structures could not be evaluated very well due to the low position of the head behind the pubic bone. Family history was negative for scalp defects, cardiac malformations or anomalies involving extremities.

Physical examination instantly after birth revealed a large scalp defect, 14 x 10 cm, over the vertex from the frontal bone extending to the parietal bones on both sides. There was a matching underlying skull ossification defect and dura defect, allowing visualization of the brain, only covered by a thin, translucent membrane. A continuously bleeding prolaps of a parasagittal parietal part of the brain was visible through a tear in the thin membrane. The bleeding source was probably the superior sagittal sinus. Further inspection revealed a (minimal) brachydactyly of the first four digits of hands and feet, and hypoplastic nails. The skin showed a cutis marmorata. Neurological examination revealed a hypotonic status on the left side of the body and less spontaneous movements on that side. The combination of ACC, TTLD,s and cutis marmorata led us to the diagnosis of AOS.

Investigation and treatment

**Figure 1:**
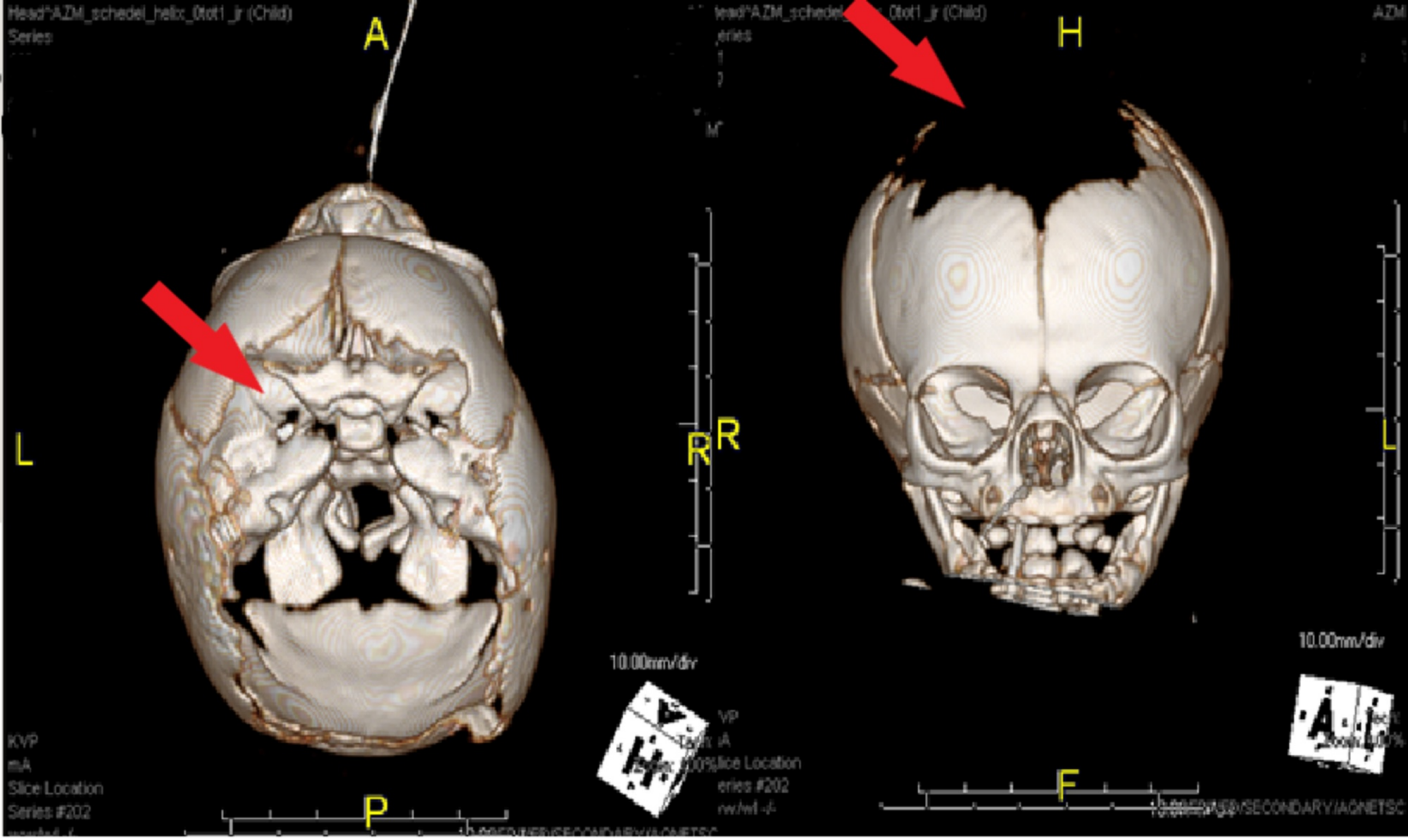
3D representation of postnatal computed tomography scan which verified the extensive defect of the scalp and skull Left image: cranial view. Right image: frontal view.

The newborn was transferred from the hospital in Roermond to our academic hospital immediately after birth, using a mobile neonatal intensive care unit. Immediately after arrival, a computed tomography (CT) scan verified the extensive defect of the scalp and skull, the herniation of brain tissue, and showed active bleeding along the falx cerebri and superior sagittal sinus (Figure 1). The CT scan also showed a traumatic fracture and molding of the existing skull.

Under continuous blood transfusion, a multidisciplinary team of paediatric anaesthesiologists, neurosurgeons, and plastic surgeons evaluated the defect and the general condition and prognosis of the infant, and decided to perform surgery on the scalp and skull defect three hours after birth. The occipital prolapse of the brain measured approximately eight cubic centimetres and was amputated up to the level of the defect. The dural defect was closed with a dural graft implant (DuraformTM, Johnson&Johnson, Codman, NJ, USA) and Surgicel Tabotamp* (Johnson&Johnson-Ethicon, NJ, USA). The skin defect was covered with Integra® Dermal Regeneration Template (Integra Life Sciences Corp., New Jersey, USA). The entire defect was wrapped with betadine antiseptic gauzes, followed by sterile gauzes and a bandage. Postoperatively, the infant was taken to the neonatal intensive care unit and underwent sterile dressing changes twice a week and was put on an intravenous prophylactic antibiotics scheme.

At approximately two weeks of age, a magnetic resonance imaging (MRI) scan of the brain was made because of a progressive herniation of the right parieto-occipital area. The MRI showed thrombosis of the sagittal sinus. Fortunately the patient did not develop venous infarctions or signs of increased intracranial pressure. Surgical treatment was started with re-removal of the brain protrusion, and Spongostan* (Johnson&Johnson-Ethicon, NJ, USA) was placed into the open ventricle, followed by suturing in an EthisorbTM Dura Patch (Johnson&Johnson, CODMAN®, NJ, USA) and temporary covering with TachoSil® Surgical Patch (Baxter international Inc., Deerfield, Il, USA). At the age of four weeks, the infant had another episode of cerebrospinal fluid (CSF) leakage and thereby a second, small defect of the right parieto-occipital area, and brain prolapse, which caused epileptic seizures. The neurosurgeon reoperated and the brain prolapse could be reduced without resection. The defect was covered with a Dura Patch and TachoSil, and the CSF gap was closed with new sutures. Postoperatively, no signs of hydrocephalus developed.

After a month of conservative treatment with dressing changes every two days, a positive pressure isolation room (pathogen free), antibiotics, antimycotics (Daktarin), and intensive care, a fourth operation was performed for debridement of the wound and to change the TachoSil. During the entire period, the patient was treated in an almost upright position to prevent pressure on the wound. Acetazolamide (Diamox) was administered in a low dosage because of the three times of recurring CSF leakage. Due to rejection of the Integra®, the last operation was performed, in combination with the construction of a delay procedure, for a planned pedicled occipital flap by the plastic surgery team, to allow definitive closure of the areas with Dura Patches after 4 to 6 weeks. Further conservative treatment with gauzes and suspicion Fucidin cream twice a week was introduced to bridge the period before definitive transposition of the occipital flap. However, after three weeks of bandage changes, the edges of the wound appeared to have reepithelialised spontaneously. It was decided to continue the conservative management plan, instead of reconstruction with the occipital flap. Conservative treatment was continued until a complete healing of the dermal defect occurred after eight months. During this period, systemic broad-spectrum antibiotics were continuously administered (i.e. amoxicillin/clavulanic acid, flucloxacillin, ceftazidime, cefazolin, and gentamicin) as well as topical application of fucidin ointment. As soon as there was any sign of fungal infection, miconazol cream was added to the topical antibiotic treatment. Further conservative management consisted of positive pressure environment, sterile wound dressing changes, and counselling for the parents and child.

Outcome and follow-up

**Figure 2:**
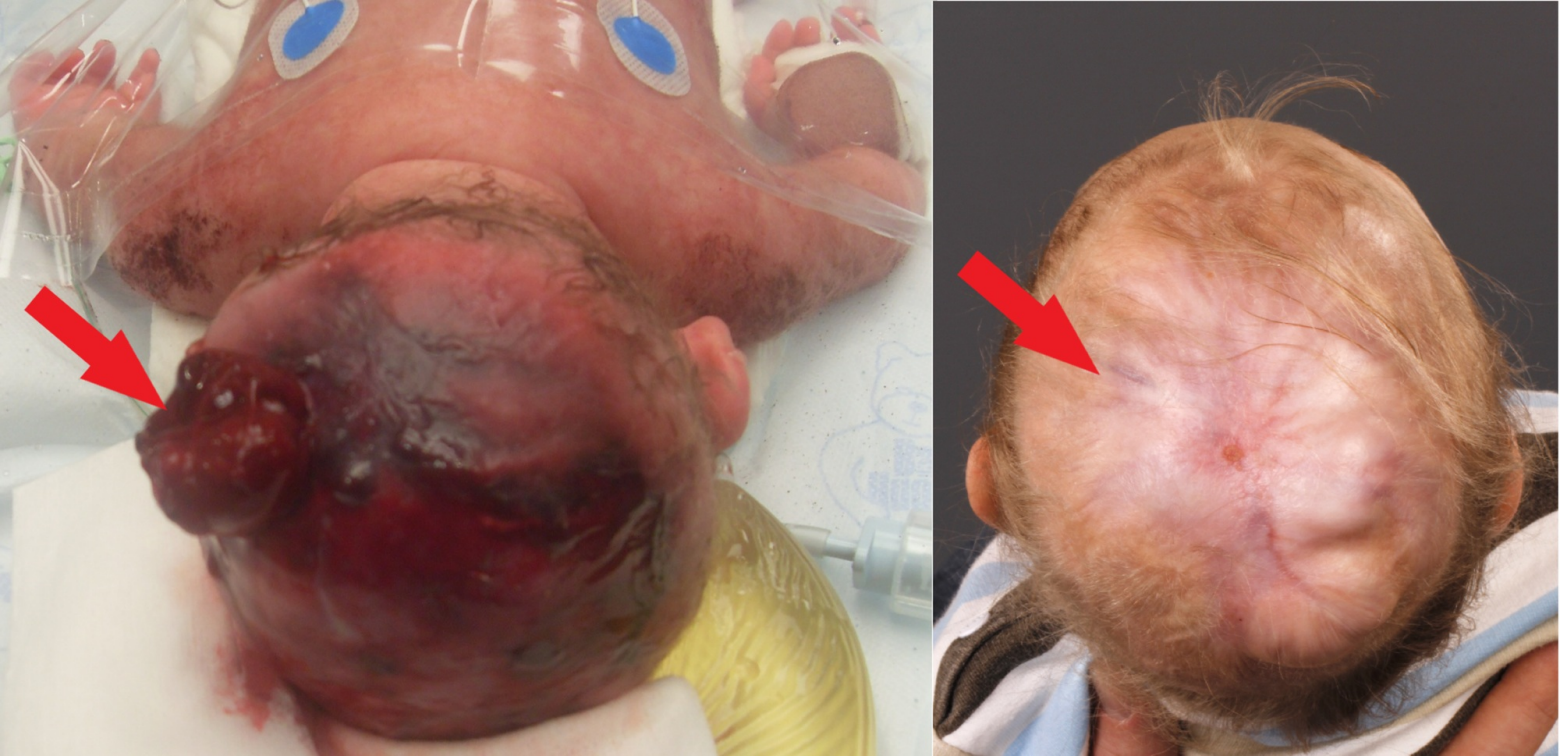
Postnatal defect and at 17 months’ age Left image: postnatal defect with brain protrusion. Right image: healed skin after conservative treatment at 17 months of age.

At the present age of 17 months, the child is in good physical condition. Clinical evaluation reveals stable growth retardation (P<5) and a three-month development delay in comparison with infants of his age. Neurologically, the child is moving symmetrically, there is no evidence of neurological deficit. By physical examination, a defect central on the skull, covered by a completely healed skin is still palpable, located at the area of the initial tear of the thin membrane, where the dura patch was introduced (Figure 2). Interestingly, the rest of the scalp defect shows spontaneous ossification. Until the bony skull defect is completely healed, the patient will wear a helmet to prevent any accidental injury.

## Discussion

To our knowledge, this case is the most extensive case of aplasia cutis with underlying skull defect described in the literature.

Management of skull defects resulting from cutis aplasia remains controversial. Both surgical intervention and conservative management, or a combination have been described [12,13]. The suggested treatment strategy is dependent on the dimension of the skull defect and the overall condition of the infant. Surgical management is not considered to be a standardised treatment for ACC. Ideally, we try to achieve the following objectives as mentioned by Albright et al. [20]:

- protection of the underlying brain and dural venous sinuses by keeping the laesion moist using sterile saline-soaked gauzes. This prevents infection and avoids desiccation and cracking of exposed tissue overlying the dural venous sinus;

- eventual healing or repair of any underlying skull defect;

- coverage of the head with hair-bearing scalp;

- minimisation of scar-tissue;

- avoidance of surgical complications/iatrogenic trauma;

- minimisation of hospital days;

- minimisation of total treatment course.

In this unique case, we have demonstrated that it is possible to finish a treatment conservatively even in case of a large congenital skull defect caused by ACC. We have followed a stepwise approach in which we first excised the brain herniation, and covered the membrane with a Dura Patch and a dermal skin substitute (Integra®). Due to rejection of the Integra®, we planned a delayed, pedicled occipital flap. However, conservative management led to early reepithelialisation, skin growth, and ossification of the thin membrane. Therefore, the pedicled flap did not have to be used.

In order to reach a satisfactory result with conservative measures, and to avoid serious complications--such as meningitis, hyponatremia with seizures or brain herniation, or massive haemorrhage--treatment should be carried out under strict conditions. We recommend a positive pressure isolation room, dressing changes every second day, topical and systemic antibiotics until complete skin healing is reached, antimycotics, and intensive care conditions. Diamox can be considered to help prevent CSF leakage.

An important aspect of the treatment approach was the continuous administration of antibiotics to avoid potentially fatal consequences associated with cerebral infection. A multidisciplinary approach is preferred to provide the best (conservative) treatment for a large skull defect in cutis aplasia congenita, and to avoid serious complications.

## Conclusions

To our knowledge, this case is the most extensive case of ACC with underlying skull defect described in the literature, and this article reports the possibility of finishing a treatment conservatively even in case of a very large (14 x 10 cm) skull defect. An important aspect of the treatment approach was the continuous administration of antibiotics to avoid potentially fatal consequences associated with cerebral infection. A multidisciplinary approach is preferred to provide the best (conservative) treatment for a large skull defect in cutis aplasia congenita and to avoid serious complications.
